# Supplementary material for: Aberrantly Expressed RECQL4 Helicase Supports Proliferation and Drug Resistance of Human Glioma Cells and Glioma Stem Cells
Source: Cancers (Basel). 2020 Oct 11;12(10):2919. doi: 10.3390/cancers12102919 (PMC7650617; doi:10.3390/cancers12102919)
Supplement: Supplementary file 1 [file cancers-12-02919-s001.zip › cancers-925505-supplementary.docx]

Article

Aberrantly expressed RECQL4 helicase supports proliferation and drug resistance of human glioma cells and glioma stem cells

Sylwia K. Król, Agnieszka Kaczmarczyk, Kamil Wojnicki, Bartosz Wojtas, Bartłomiej Gielniewski, Wieslawa Grajkowska, Katarzyna Kotulska, Cezary Szczylik, Ryszard Czepko, Mariusz Banach, Wojciech Kaspera, Wojciech Szopa, Andrzej Marchel, Tomasz Czernicki and Bozena Kaminska

Supplementary Materials

**Table S1.** Sequences of primers for qPCR.

| **Gene** | **Primer** | **Sequence** |
| --- | --- | --- |
| *GAPDH* | forward | 5’-TCCTGGAACAGCAAAACAAG-3’ |
|  | reverse | 5’-CAGCCTCAGGTTGGTTTCAT-3’ |
| *RECQL4* (1) | forward | 5’-GAGGAGGCCATCGAGACTTT-3’ |
|  | reverse | 5’-GTATAGGTGGTCGCCAGCAG-3’ |
| *RECQL4* (2) | forward | 5’-GGATCCTGTCTGGCATCTCC-3’ |
|  | reverse | 5’-GGAGAGACGACCAACGTGAG-3’ |
| *NANOG* | forward | commercially available from Qiagen (Hilden, Germany) QT01844808 |
|  | reverse |  |
| *SOX2* | forward | 5’-GGGGAAAGTAGTTTGCTGCC -3’ |
|  | reverse | 5’-CGCCGCCGATGATTGTTATT-3’ |
| *PROMININ-1* | forward | 5’-TGGATGCAGAACTTGACAACGT-3’ |
|  | reverse | 5’-ATACCTGCTACGACAGTCGTGGT-3’ |
| *POU5F1* | forward | commercially available from Qiagen (Hilden, Germany) QT00210840 |
|  | reverse |  |

**Table S2.** Antibodies for Western blot.

| **Protein** | **Company** | **Cat. No.** | **Host** | **Dilution** |
| --- | --- | --- | --- | --- |
| RECQL4 | Cell Signaling | #2814 | rabbit | 1:1000 |
| RECQL4 | Novus Biological | #25470002 | rabbit | 1:1000 |
| cleaved PARP1 | Cell Signaling | #9541S | rabbit | 1:1000 |
| cleaved caspase 3 | Cell Signaling | #9661S | rabbit | 1:1000 |
| cleaved caspase 7 | Cell Signaling | #9491S | rabbit | 1:1000 |
| phospho-H2AX | Abcam | ab-26350 | rabbit | 1:1000 |
| Aurora kinase B | Bethyl Laboratories | #A300-431A | rabbit | 1:1000 |
| phospho-CHK1 (Ser345) | Cell Signaling | #9947 | rabbit | 1:1000 |
| phospho-CHK2 (Thr68) | Cell Signaling | #9947 | rabbit | 1:1000 |
| phospho-p53 (Ser15) | Cell Signaling | #9286 | mouse | 1:1000 |
| TOMM20 | GeneTex | GTX133756 | rabbit | 1:500 |
| β-Actin | Sigma-Aldrich | #A3854 | mouse | 1:30000 |
| GAPDH | Millipore | #MAB374 | mouse | 1:1000 |

Antibodies were diluted in a TBS-T (or 5% BSA in TBS-T or 5% non-fat milk in TBS-T).

Adherent and sphere cultures, treatments

Human glioma cell lines LN18, LN229, U87-MG and U251 were from American Type Culture Collection (ATCC, Manassas, VA, USA). Cells were cultured as described (20). GBM patient-derived glioma primary cultures WG4 and IPIN20160420 were generated as described (20) and cultured in DMEM/Nutrient Mixture F-12, GlutaMAX™ medium, supplemented with 10% FBS (Gibco Life Technologies, Rockville, MD, USA) and antibiotics (100 U/mL penicillin, 100 µg/mL streptomycin). Normal human astrocytes (NHA, Lonza Walkersville, MD, USA) were cultured in a commercial medium as described (33). All cell lines were *Mycoplasma sp*. free. Media were replaced every 3 days and cultures were maintained in a humidified atmosphere of 5% CO_2_ and 95% air, at 37°C. Temozolomide (TMZ, Sigma-Aldrich, Munich, Germany) was dissolved in water, Olaparib (OLA, MedChemExpress, Monmouth Junction, NJ, USA) was dissolved in DMSO.

For sphere cultures, LN18 glioma cells were seeded at a low density (1500 cells/cm^2^) on non-adherent plates and cultured in DMEM/ F-12, GlutaMAX™ supplemented with 2% B27 (Gibco Invitrogen, Basel, Switzerland), 20 ng/mL rhuman bFGF (Miltenyi Biotec, Bergisch Gladbach, Germany), 20 ng/mL rhuman EGF (StemCell Technologies, Vancouver, BC, Canada), 0.0002% heparin (StemCell Technologies, Vancouver, BC, Canada) and antibiotics (Gibco Invitrogen, Basel, Switzerland). Cells were fed every 3 days by adding 1 mL of the fresh medium. Spheres were collected by centrifugation at 1200 rpm at +4°C for RNA and protein isolation.


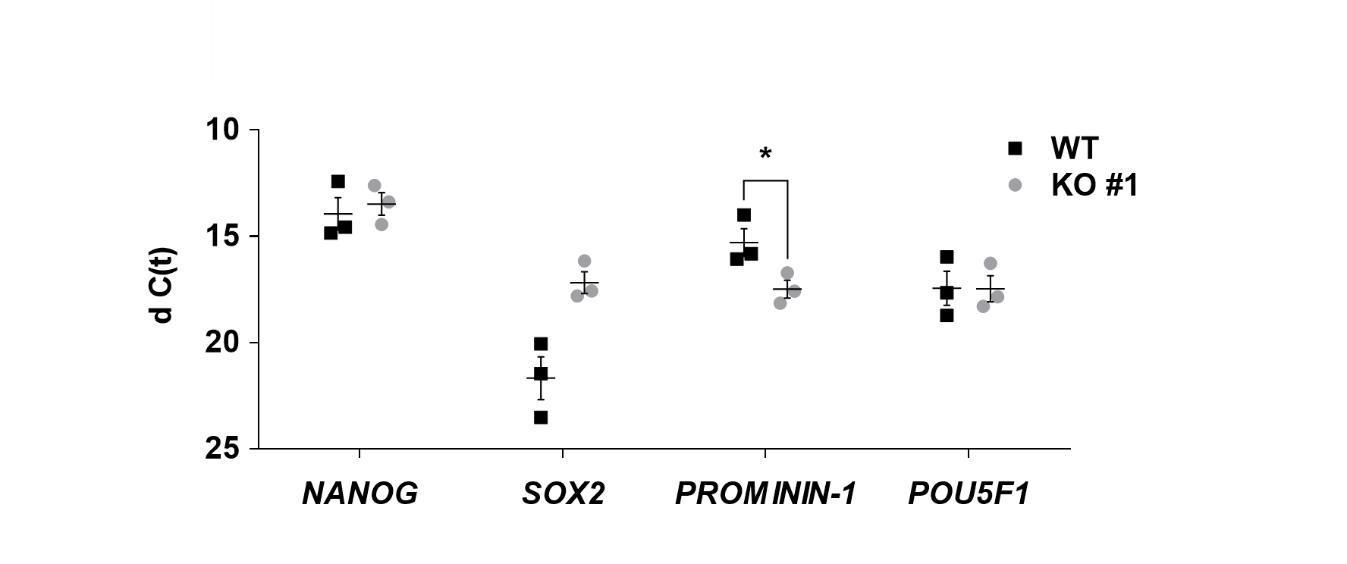


**Figure S1.** Expression of stemness genes in glioma spheres cultures. Quantitative analysis of the expression of selected stem cell markers at mRNA level in WT and RECQL4 KO LN18 glioma spheres. The expression was normalized to *GAPDH* used as an internal reference gene. Results represent means ± SEM (n=3). Statistical significance was determined by two-tailed paired t-test. P values were considered as significant when * *p* < 0.05.

| **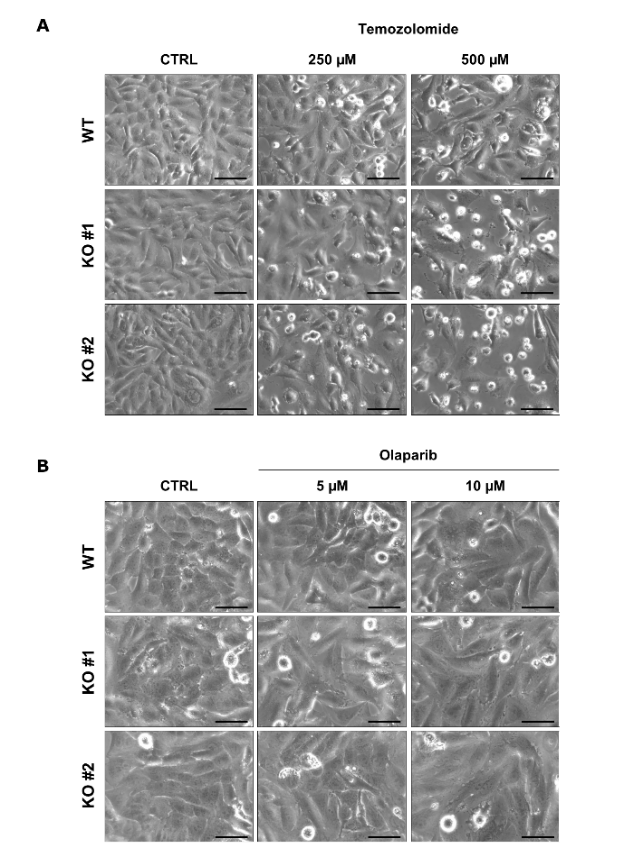** | **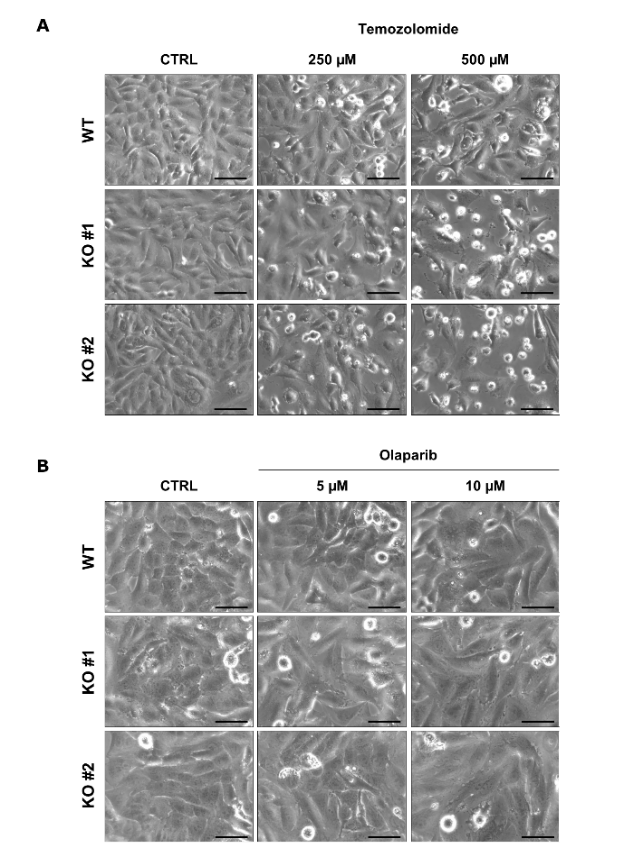** |
| --- | --- |

**Figure S2.** LN18 glioma cells exposed to chemotherapeutics**.** (**A**)**.** Representative images showing the effects of TMZ treatment (250, 500 µM for 72h) on morphology of WT and RECQL4 KO LN18 cells. (**B**)**.** Representative images showing the effects of olaparib treatment (5, 10 µM for 72h) on morphology of WT and RECQL4 KO LN18 cells. Scale bar represents 50 µm.


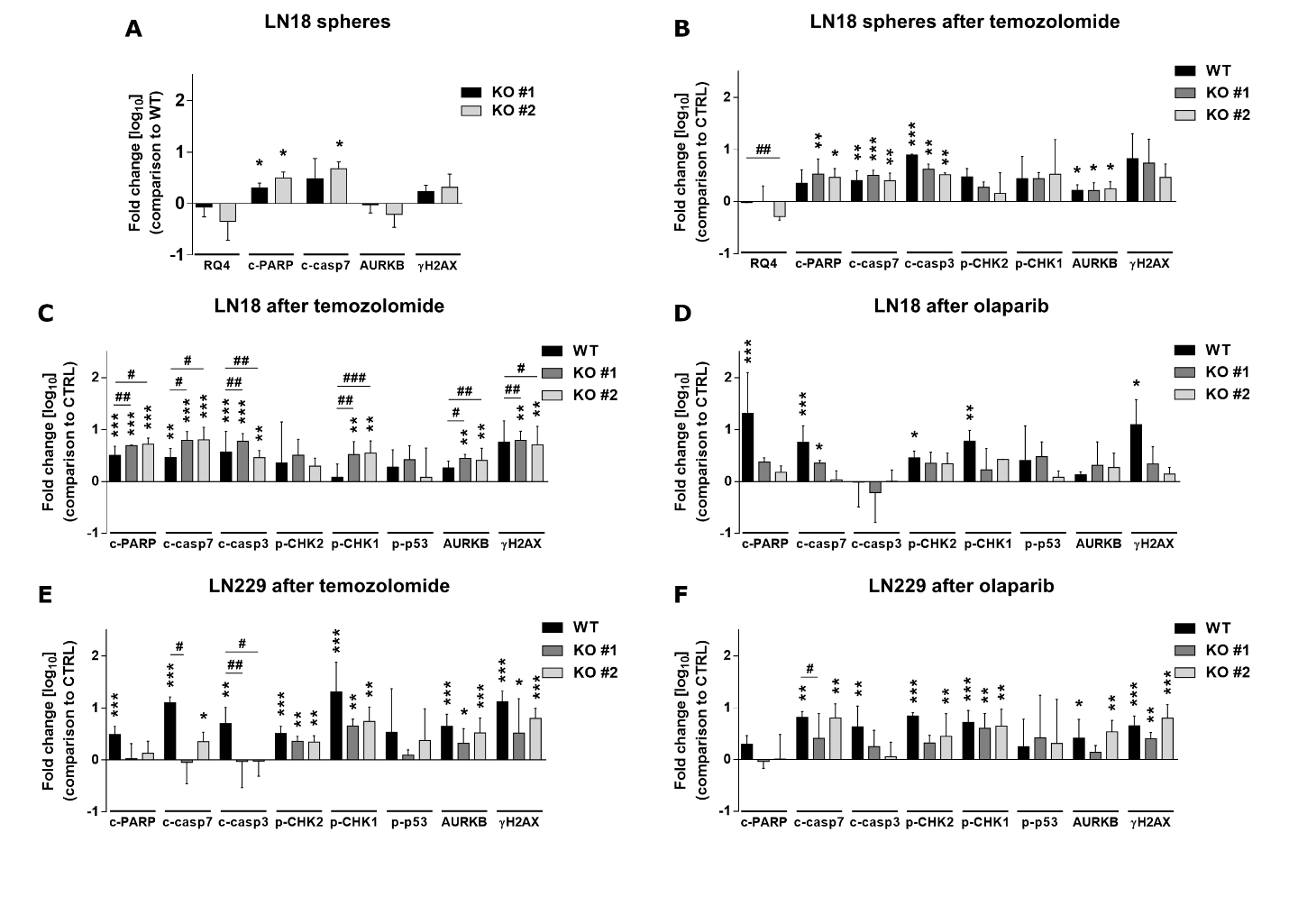


**Figure S3.** Densitometric analysis of Western blot. Densitometry analysis of immunoblots from RECQL4 KO cells. Levels of specific proteins were first related to β-ACTIN levels in a given sample and then expressed as fold change of a given control. (**A**). Protein levels in WT and RECQL4 KO cells growing as spheres. (**B-F).** Levels of proteins in cells treated to 250 µM temozolomide (TMZ) (**B,C,E**) or 10 µM olaparib (**D,F**) Statistical significance was determined by one-way or two-way ANOVA followed by Dunnett’s post hoc test. Statistical significance in comparison to control cells (CTRL) was depicted as a “*” and differences between the WT and RECQL4 KO cells were shown by “#”. The results represent the means ± SD (*n* ≥ 2). P values were considered as significant when * *p* < 0.05, ** *p* < 0.01, *** *p* < 0.001.
